# Current Status and Challenges of Local Resection for Early Gastric Cancer in East Asia

**DOI:** 10.3390/cancers18121885

**Published:** 2026-06-09

**Authors:** Shinichi Kinami, Yasuto Tomita, Koichi Okamoto, Hiroyuki Takamura

**Affiliations:** Department of Surgical Oncology, Kanazawa Medical University, Daigaku, Uchinadamachi, Kahoku 920-0293, Japankokamoto@kanazawa-med.ac.jp (K.O.);

**Keywords:** early gastric cancer, local resection, sentinel node biopsy, function-preserving gastrectomy, delayed gastric emptying

## Abstract

Background/Objectives: Standard gastrectomy with lymph node dissection up to D1+ achieves good oncologic control for early gastric cancer not amenable to endoscopic submucosal dissection, yet it frequently leads to post-gastrectomy syndromes and long-term nutritional impairment. Local resection of the stomach reduces post-gastrectomy syndrome; however, the extent of lymph node dissection should be limited beyond D1+ in such cases. This review evaluates the safety of local resection for early gastric cancer reported in East Asia. Methods: We reviewed current concepts and clinical evidence regarding (i) the limitations of preoperative nodal staging, (ii) sentinel node biopsy and function-preserving gastrectomy, and (iii) functional outcomes and procedure-specific complications following local resection, with a focus on delayed gastric emptying. Results: Conventional imaging and biomarkers are inadequate for reliable preoperative identification of node-negative disease. Conversely, sentinel node biopsies demonstrate high intraoperative diagnostic accuracy. Large prospective studies have revealed that, when indications are strictly adhered to, sentinel node biopsy-guided function-preserving gastrectomy can yield survival outcomes comparable to those of standard gastrectomy. The indications for local resection include solitary submucosal tumors below 4 cm in size, diagnosed as node-negative by sentinel node biopsy. Although the available quality-of-life data are generally favorable, there is risk of delayed gastric emptying in local resection with limited lymph node dissection in cases of early gastric cancer. Postoperative gastric deformity following closure was identified as the primary cause. Conclusions: Local resection for submucosal gastric cancer guided by sentinel node biopsy may be oncologically acceptable and function-preserving; however, the prevention of gastric deformity is crucial for its safe implementation.

## Simple Summary

Standard gastrectomy with lymph node dissection for early gastric cancer typically achieves good oncologic control; however, post-gastrectomy syndromes and long-term nutritional impairment may ensue. In early gastric cancer, the risk of nodal metastasis is relatively low, suggesting that local resection may be feasible for carefully selected patients. Sentinel node biopsy can facilitate the intraoperative diagnosis of node-negative cases. This review synthesizes current evidence on local resection guided by sentinel node biopsy in East Asia and discusses an important complication: delayed gastric emptying. This complication may be related to postoperative stomach deformities and can be mitigated through meticulous closure techniques.

## 1. Introduction

The standard curative surgical approach for gastric cancer involves a wide-area gastrectomy with lymph node dissection [1]. Although lymph node dissection has been a component of gastric cancer surgery since its inception [2], its necessity has been debated owing to its invasiveness, high morbidity, and in-hospital mortality rate [3,4,5]. Nevertheless, prospective clinical trials have demonstrated its effectiveness in improving long-term survival, thereby establishing lymph node dissection up to D2 as the standard treatment for locally advanced gastric cancer [6]. However, nodal dissection up to D2 sacrifices the majority of blood vessels supplying the stomach, resulting in extensive resection of the stomach, necessitating distal or total gastrectomy [1]. Such extensive gastrectomy is associated with decreased food intake, weight loss, post-gastrectomy complications (such as anemia, osteoporosis, and reflux esophagitis), and a high rate of post-gastrectomy syndromes (including dumping, early satiety, and diarrhea.) [7,8,9,10].

Unlike in locally advanced gastric cancer, the lymph node metastasis rate is low in early gastric cancer [11,12,13,14]; therefore, lymph node dissection up to D2 is excessively invasive [13,14]. Omitting lymph node dissection reduces the extent of gastric resection and can prevent and mitigate postoperative complications and symptoms [13,14]. The nodal metastasis rate in early gastric cancer, where invasion is confined to the mucosal layer, is approximately 2% [13,14]. Currently, most patients are treated with endoscopic submucosal dissection (ESD) [1]. Conversely, the nodal metastasis rate for submucosal cancer is 15–20%, which cannot be ignored [13,14]. ESD for submucosal cancers lacks curative potential. Therefore, lymph node dissection up to D1+, omission of nodal dissection of #10, #11, and #12a from D2, are recommended for submucosal cancer [1]. Function-preserving surgeries such as pylorus preserving gastrectomy (PPG) and proximal gastrectomy (PG), have also been attempted [1]. PPG is associated with a lower incidence of dumping syndrome than distal gastrectomy [15,16,17]. Additionally, PG reduces various postoperative complications compared to total gastrectomy [18]. However, their function-preserving effects are not always significant [14]. To reduce postoperative syndrome after gastrectomy, the extent of lymph node dissection should be limited beyond D1+, with the aim of local resection (LR) of the stomach [14].

LR of the stomach involves partial removal of the entire stomach wall, followed by closure using sutures [1,19]. This procedure is referred to as wedge resection [19]. Typically, mucosal resection is not included. According to the Japanese Gastric Cancer Treatment Guidelines, LR for gastric cancer is defined as “non-circumferential resection of the stomach” and is considered an experimental treatment [1]. Previously, laparoscopic LR using the lesion-lifting method was frequently performed for lesions with minimal potential for lymph node metastasis [19]. However, with the advent and widespread use of endoscopic submucosal dissection (ESD), LR for gastric cancer has nearly disappeared. Currently, LR is employed only when ESD is not an option due to technical difficulty [19].

Recent advancements in gastrointestinal endoscopy technology have led to the development of new LR techniques such as laparoscopic endoscopic cooperative surgery (LECS) [20] and endoscopic full-thickness resection [21,22], which are now being applied in clinical practice. These techniques are predominantly employed for LR in cases of gastric gastrointestinal stromal tumors (GISTs); however, they may be applicable as treatments for early gastric cancer [19].

This review evaluates the suitability of LR for early gastric cancer, considering its indications and potential challenges.

## 2. Survival Prognosis and Indication of Local Resection for Gastric Cancer

To perform LR, it is essential to preserve the numerous perigastric arteries and veins that supply the stomach to maintain adequate blood flow. However, because the perigastric lymph nodes are located around the main gastric artery, dissection requires resection of the main gastric artery. Consequently, applying LR to gastric cancer involves omitting some or all lymph node dissections compared to standard nodal dissection, which presents a significant challenge for LR in gastric cancer.

To address this issue, it is essential to improve the diagnostic accuracy of lymph node metastasis and accurately identify cases in which lymph node dissection is unnecessary because of negative metastasis.

However, preoperative diagnosis of cases with negative lymph node metastasis remains extremely challenging. Many regional stomach lymph nodes are small, and nodal metastasis in small nodes is common in gastric cancer [23,24,25]. Although computed tomography (CT) can detect macroscopic metastases, accurate diagnosis of node-negative cases remains difficult [24,25,26,27]. Magnetic resonance imaging (MRI) has historically been a reliable method for diagnosing metastasis in the past [28]; however, the development of important MRI contrast agents has been discontinued, rendering them unavailable. Notably, nodal metastases cannot be detected using serological or genetic diagnosis [13,29,30,31,32,33].

Lymph node metastasis can be diagnosed intraoperatively. Sentinel lymph node biopsy is the most effective method for diagnosing metastasis [13]. A sentinel node (SN) is defined as a node that receives lymphatic flow directly from the primary tumor [34]. Cancer metastasis is thought to progress from micrometastases to the SN. SN biopsy (SNB) to assess the presence or absence of metastasis is the most sensitive and accurate method for diagnosing lymph node metastasis [13].

Gastric cancer is one of the most common gastrointestinal cancers for which SNB has been studied [13,35,36,37,38,39,40,41,42,43,44]. Significant misunderstandings among many old researchers have plagued research on sentinel lymph node biopsy for gastric cancer, leading to a period where the very existence of the sentinel node concept was questioned [45]. A successful sentinel lymph node biopsy requires proper adjustment of six elements: an appropriate indication, an appropriate tracer selection, an appropriate tracer administration method, an appropriate identification of tracer-uptake lymph nodes, an appropriate sentinel lymph node biopsy technique, and an appropriate intraoperative rapid diagnosis method for nodal metastasis [13,14]. These elements apply to sentinel lymph node biopsies for all types of cancer. False-positive cases in older studies occurred because these elements were not properly adjusted. The JCOG trial [40] was deemed a negative study due to not only intraoperative diagnosis of metastasis, but also significant flaws in tracer selection, tracer administration method, identification of tracer-uptake lymph nodes, and sentinel lymph node biopsy technique. Numerous studies have shown that five of the six elements have been correctly identified in gastric cancer. Appropriate indications include clinical T1N0; appropriate tracers include the combined administration of blue dye and radioactive colloid; appropriate administration methods include submucosal injection using endoscopy; identification of tracer-uptake lymph nodes is performed on an ex vivo back table using tissue dissected in a specific area; and the biopsy should be performed using either lymphatic basin or sentinel basin dissection [13,14]. Experts agree on these points [46,47,48,49,50,51,52]. Unfortunately, however, there is still no consensus on appropriate intraoperative rapid metastasis diagnostic methods [13]. In recent years, indocyanine green fluorescence mapping has emerged as a viable alternative to the gold-standard combination mapping method [13,14,43,52].

The diagnostic accuracy of lymph node metastasis using combination mapping with radioisotopes and dyes or indocyanine green fluorescence is extremely high for clinically N0 early gastric cancer [13,46]. If SNB can be used to omit lymph node dissection and preserve gastric blood flow, LR becomes possible [13,14,19]. However, a crucial question remains: Is the long-term survival prognosis of surgery guided by SNB to omit lymph node dissection and reduce the extent of gastrectomy (sentinel node navigated function-preserving gastrectomy; SNFPG) comparable to that of standard surgery? Two large-scale nationwide trials have been conducted: the SENORITA trial in South Korea [47,48,49,50,51] and a prospective trial by the Japanese Society of Sentinel Node Navigation Surgery [52]. The SENORITA trial aimed to verify the non-inferiority of 3-year disease-free survival between laparoscopic standard gastrectomy and laparoscopic SNB-guided surgery (LSNNS). Unfortunately, noninferiority was not demonstrated [49]. This may be attributable to recurrent gastric cancer and metachronous multiple gastric cancers being treated together without distinction. As the LSNNS group had a larger residual gastric area, it is natural that there were more metachronous multiple gastric cancers [53]. In the SENORITA study, the 5-year disease-free survival rates were 80.1% in the laparoscopic standard gastrectomy group and 88.9% in the LSNNS group [50]. There were no significant differences in the overall survival, disease-free survival, or disease-specific survival between the two groups, and all ten cases with metachronous multiple gastric cancers were cured with additional treatment. A prospective trial in Japan [52] has not yet been published, but at ASCO 2026, its results were reported, demonstrating the non-inferiority of SNFPG.

Therefore, it is concluded that SNFPG may exhibit a life expectancy comparable to that of standard surgery, with its curative efficacy potentially uncompromised, even with a reduced extent of dissection, as indicated by SNB. Furthermore, in a single-center study, Kinami et al. compared the prognosis of guideline surgery cases and SNFPG cases, limiting them to early gastric cancers located in the middle and lower region [54]. They reported that the overall survival of 123 patients with SNFPG was significantly better than that of 300 patients who underwent guideline surgery and that this difference was due to deaths from other diseases, particularly respiratory diseases. Stomach-preserving treatments have the potential to improve life expectancy by reducing deaths from other diseases, maintaining nutritional status, and preventing respiratory complications [13,19,54].

Consequently, it can be concluded that LR is indicated for early gastric cancer cases with intraoperative SNB metastasis negativity [13,19]. However, favorable outcomes cannot be achieved with gastric cancer SNB if appropriate indications are not followed [13,14,54]. According to the Japanese SNNS Study, the indication for gastric cancer SNB is a solitary submucosal tumor that is less than 4 cm in size and shows no obvious lymph node enlargement on CT [13,14,46] (Table 1).

## 3. Special Characteristics of Local Resection for Gastric Cancer

As previously highlighted, ESD is now the standard treatment [14,19,55,56,57,58,59]. Therefore, the concomitant use of SNB is essential when performing LR for early gastric cancer [19]. However, SNB for gastric cancer differs from that for breast cancer in unique ways [13,19,46,54].

SNB is the most frequently used standard treatment for breast cancer. SNB is primarily used to treat breast cancer with no macroscopic metastasis [60]. It is mainly used to indicate the omission of axillary lymph node dissection and prevent lymphedema in the affected arm [60]. For breast cancer, blue dye, indocyanine green (ICG), and radioisotope colloids are commonly used as tracers [60,61,62,63]. Combination mapping is employed, and tracer uptake in the lymph nodes is detected using gamma probes or ICG fluorescence detectors. A small incision is made in the axilla, and only SNs are biopsied. One to three SNs are obtained and metastasis is assessed using multiple sections spaced 2 mm apart. Even if a false-negative result occurs in the rapid intraoperative diagnosis, axillary lymph node dissection can be performed later because the axillary lymph nodes are close to the body’s surface. Furthermore, the emphasis on ultrastaging has become more significant than the criterion for omitting axillary lymph node dissection; therefore, the need for rapid intraoperative determination of SN metastasis has decreased [64,65].

In contrast, gastric cancer has a high median number of SNs (six) [14,54]. In addition, there is no room for delayed re-dissection. Therefore, rapid pathological diagnosis of nodal metastasis is difficult in gastric cancer because many lymph nodes must be assessed within the limited timeframe of surgery [14,54]. Currently, metastasis in gastric cancer SNB is determined by rapid pathological diagnosis of the largest central one-plane sections [46]. However, considering the feasibility, safety, objectivity, uniformity, and standardization of diagnosis, this should be changed to a genetic diagnosis [13,14,54]. In addition, there are concerns regarding curative efficacy without concurrent backup dissection [14,54]. Research on the genetic diagnosis of lymph node metastasis is ongoing and evidence remains limited. Conversely, backup dissection has already been performed and the results are known. In the SENORITA trial, a backup dissection called sentinel basin dissection was performed. This method involves *en bloc* dissection of the area containing the tracer-incorporating lymph nodes with a 1 cm margin [47,48,49,50,51]. Of the 198 patients in the SENORITA LSNNS group, one had lymph node recurrence and two had distant metastatic recurrence [51]. In the Japanese SNNS trial, a backup dissection, called lymphatic basin dissection, was performed concurrently. This method involves *en bloc* dissection of the lymphatic drainage area from the gastric wall where the tumor resides to the distal SN [46]. Although no direct comparison has been made, it is presumed that the Japanese lymphatic basin dissection method has a wider dissection range than the Korean sentinel basin dissection method. Kinami et al. reported no lymph node recurrence in 190 cases of function-preserving surgery combined with lymphatic basin dissection [66].

**Figure 1:**
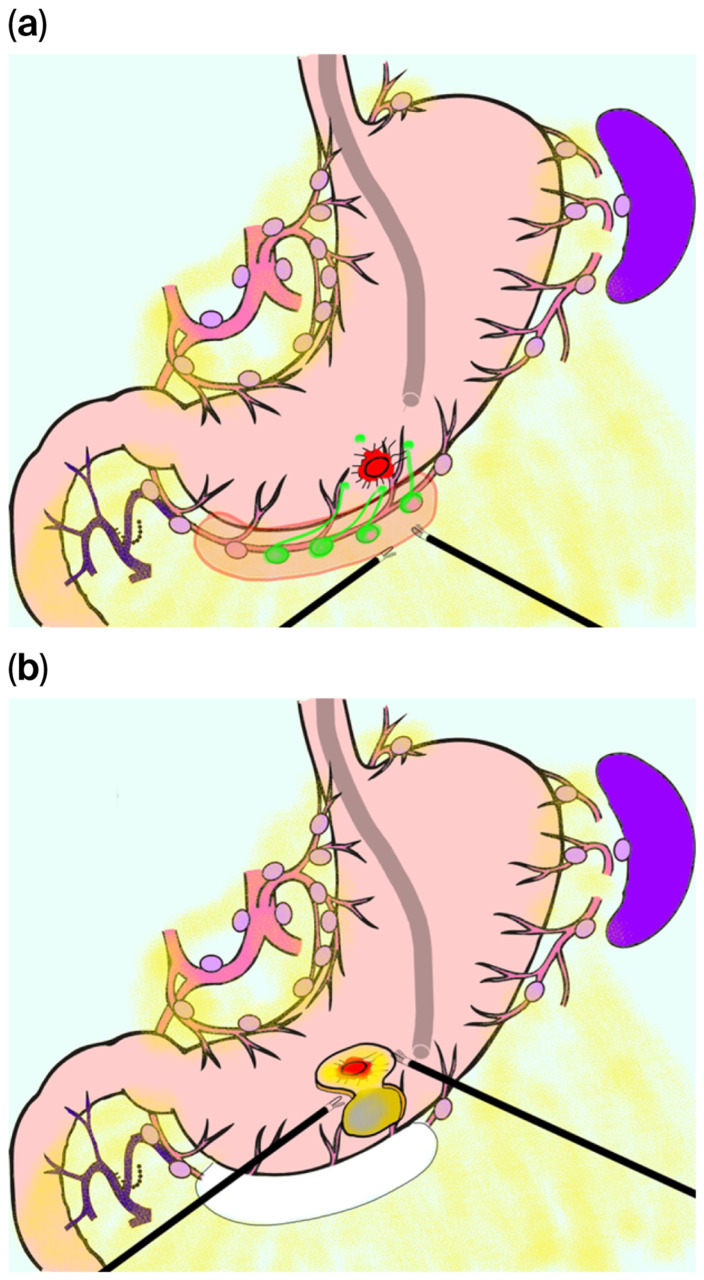
**Local resection with lymphatic basin dissection for the submucosal cancer located at the greater curvature.** (**a**) Lymphotropic tracers are administered to the submucosa surrounding the lesion, and a sentinel lymph node biopsy is performed. In the present case, because the sentinel lymph nodes are located only within the No. 4d region, lymphatic basin dissection of the right gastroepiploic artery basin is performed. (**b**) If no nodal metastases are found on sentinel lymph node biopsy, local resection is performed. Using the LECS method, full-thickness resection is performed using an endoscopic submucosal dissection device to minimize the resection area, and the site is closed laparoscopically with suturing.

**Figure 2:**
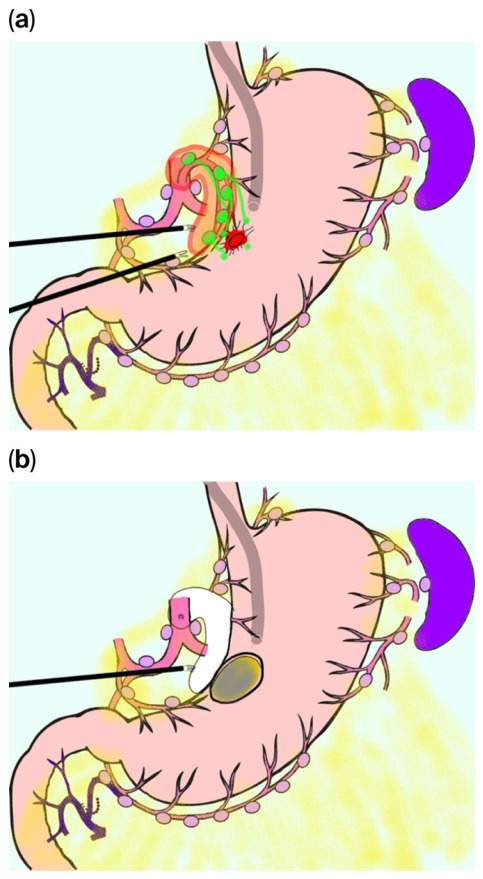
**Local resection with lymphatic basin dissection for the submucosal cancer located at the lesser curvature.** (**a**) In this case, lymphotropic tracers flow to the only left gastric artery basin, and the sentinel lymph nodes are located only within the No. 3a and No. 7 region; therefore, lymphatic basin dissection of the left gastric artery area should be performed. (**b**) Unlike the case presented in Figure 1, this procedure requires sacrifice of the celiac and gastric branch of the vagus nerve because of the lymphatic basin dissection performed in the left gastric artery basin.

Therefore, unlike that for GIST, LR for early gastric cancer involves dissecting a certain range of perigastric nodes [19] (Figure 1). This results in the resection of some of the perigastric autonomic nerves. This is particularly problematic when the lesion occupies the lesser curvature, as resection of the gastric branch of the vagus nerve on the lesser curvature side occurs alongside lymph node No. 3a dissection (Figure 2). Further details are discussed herein.

## 4. Functional Preservation in Local Resection for Gastric Cancer

It is crucial to evaluate the functional aspects of patients after LR for gastric cancer, such as food intake volume capacity and incidence of postgastrectomy syndrome. This is because, even if LR for gastric cancer without nodal metastasis does not affect the prognosis of cancer treatment, adopting LR is not beneficial if functional preservation is not achieved [19].

Analysis of the previously cited PGSAS study showed that functional scores for LR were higher than those for standard surgery and guideline-based function-preserving gastrectomies such as PPG and PG [67]. However, many LRs in this trial were for GISTs, and lymph node dissection was not performed. Therefore, the results of this study should be interpreted with caution.

The SENORITA trial investigated the EORTC-QLQ-C30 and STO22 [51]. Compared with the standard gastrectomy group, the LSNNS group showed higher BMI, hemoglobin, and albumin levels, as well as improved physical function, dyspnea, appetite loss, dysphagia, eating restrictions, anxiety, taste changes, and body image [51]. Of the 194 patients in the LSNNS group, 165 (85%) underwent LR, suggesting that patients who underwent LR had a higher quality of life (QoL) than those who underwent standard gastrectomy.

Furthermore, although it was a single-center study, Okubo et al. reported that LR using SNB as an indicator was associated with better weight retention, lower gastroesophageal reflux disease score, and less residual gastritis than standard surgery [68]. They also reported that the PGSAS-45 dyspepsia subscale, amount of food per meal, need for additional food, and dissatisfaction with food and daily life improved significantly better [68].

Although the currently available data are promising, it remains difficult to determine whether the incidence of postgastrectomy symptoms and QoL in patients undergoing LR are truly superior to those in patients undergoing standard surgery until the results of the Japanese SNNS trial become available. Because the SNNS trial also includes a QoL assessment using the EORTC, its findings will likely provide more definitive evidence.

## 5. Complications Specific to Local Resection

LR for gastric cancer, when limited to metastasis-negative cases diagnosed by SNB, is an effective method that can reduce postgastrectomy syndrome without compromising the survival prognosis. However, there is limited experience with this surgical procedure, and many aspects remain unclear. This review addresses the currently suspected complications.

Harada et al. reported that among 213 LECS cases, Clavien–Dindo grade II or higher complications occurred in six cases, including one case of postoperative bleeding, one case of anastomotic leakage, and four cases of delayed gastric emptying (DGE) [69]. Hashimoto et al. reported that among 201 LECS cases nationwide, Clavien–Dindo grade II or higher complications occurred in 10 cases, including three cases of postoperative bleeding, two cases of bacteremia, and one case each of anastomotic leakage, surgical wound infection, hypertension, urinary tract infection, and DGE [70]. Other reports have described postoperative bleeding and suture ulcers as complications of local gastrectomy [71]. Some reports have also indicated recurrence at surgical margins and metachronous multiple cancers [72].

These reports suggest that the postoperative complications of LR without lymph node dissection include postoperative bleeding and anastomotic ulcers, whereas DGE and metachronous multiple gastric cancers are considered specific long-term complications after LR.

The postoperative bleeding described here is intraluminal bleeding from the suture line due to abundant blood flow in the gastric wall, and not intraperitoneal bleeding. Proper control of bleeding from the resection line before gastric suturing can prevent bleeding. Even if bleeding occurs, it can be controlled endoscopically. Preventing anastomotic ulcers is crucial and requires the perioperative suppression of acid secretion (via proton pump inhibitors or H2 blockers) and adequate blood flow to the stomach. Metachronous multiple gastric cancers are common after function-preserving surgeries. The incidence of metachronous multiple gastric cancers increases with the extent of the remaining gastric mucosa. Its incidence is approximately 8% after segmental gastrectomy and LR [53]. However, ESD for metachronous multiple cancers is relatively easy because the remaining stomach after LR provides a sufficiently large working space for endoscopic manipulation, and these lesions can generally be managed successfully with regular endoscopic follow-up [53].

**Figure 3:**
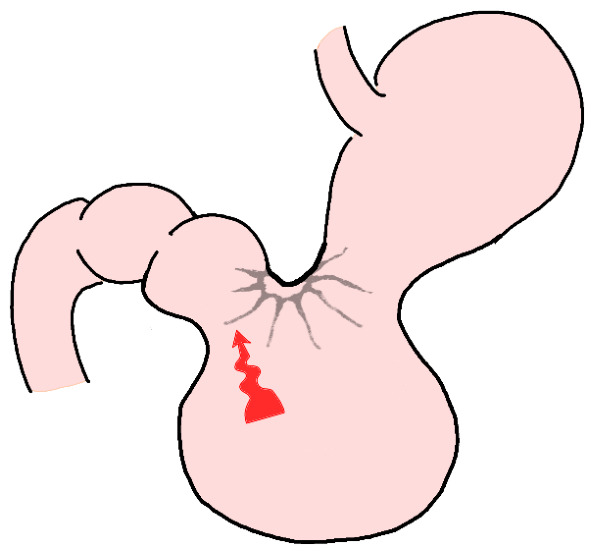
**The sac-like deformation after peptic ulcer located at the lesser curvature.** After a benign gastric ulcer located on the lesser curvature of the stomach heals, the lesser curvature shortens owing to cicatricial contraction, leading to a sac-like deformation. This causes the stomach to expand into a sac-like shape, impairing food emptying.

**Figure 4:**
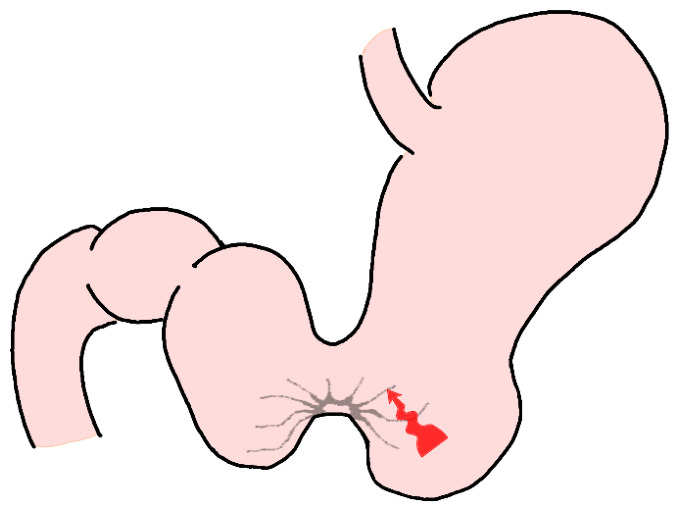
**The hourglass-shaped deformation after peptic ulcer located at the greater curvature.** After a benign gastric ulcer located on the greater curvature of the stomach heals, the greater curvature shortens owing to cicatricial contraction, leading to an hourglass-shaped deformation. This causes the stomach to expand into an hourglass shape, impairing food emptying.

Therefore, DGE is the most concerning complication of LR. DGE is characterized by delayed emptying of food from the stomach, which leads to abdominal distension, vomiting, and inability to eat [72,73,74]. DGE frequently occurs after distal gastrectomy or PPG [73,74,75] but can also occur in unresected stomachs if deformed by a gastric ulcer [76]. DGE develops when the lesser curvature shortens and becomes sac-like due to scar contraction (Figure 3) from an angular gastric ulcer or when hourglass-shaped deformation (Figure 4) occurs due to scar contraction from a body ulcer [76].

According to the aforementioned LECS study, DGE may occur in 0.5–1.8% of LR cases without lymph node dissection [69,70]. The etiology of DGE is unknown; however, it is more prevalent in older patients, women, and those with lesions in the lesser curvature or larger resections [70,77,78,79]. It has also been suggested that DGE is more prevalent in patients with LR near the pylorus [77,78,79]. DGE is likely to develop when gastric motility is reduced and the stomach morphology changes after suturing.

## 6. The Relationship Between Delayed Gastric Emptying and Limited Lymph Node Dissection

Conversely, comprehensive reports on the extent of DGE in patients with intraoperative metastasis-negative SNB diagnosis and limited lymph node dissection with LR are lacking. There is a particular concern that DGE may develop in LR cases in which lymph nodes on the lesser curvature side (No. 3a) are dissected and the gastric branch of the vagus nerve is denervated (Figure 2).

Gastric motility is preserved even after stomach denervation; however, changes occur in contractility and peristalsis. In animal studies, trunk vagotomy (TV) has been observed to decrease the contractility of the antrum and pylorus and to produce a monotonic contraction pattern in the later stages of feeding [80,81,82]. As these changes can induce DGE, it was thought that reducing pyloric resistance with pyloroplasty was necessary after TV. In contrast, in selective proximal vagotomy (SPV), which cuts only the gastric branch in the parietal cell region and preserves the hepatic, celiac, and pyloric branches, as well as the Latarjet and pyloric sinus branches, it was thought that gastric emptying is preserved, DGE does not occur, and pyloroplasty is unnecessary [83]. However, decreased contractility and monotonization of pyloric motility have been reported in the later stages of feeding, even after SPV [83,84,85,86]. Therefore, it is presumed that even in LR cases in which limited lymph node dissection of No. 3a is performed, reduced contractility and monotonization of peristalsis will occur.

However, it remains unclear whether these conditions lead to immediate DGE. Postoperative gastric motility disorders improve over time [82,87]. In experiments with dogs, Kubota et al. reported that LR of the lesser curvature caused impaired acceptance, relaxation, and motility index, but did not affect gastric emptying [88]. Kinami et al. reported that of 57 cases of LR treated with SNB, 5 (8.8%) developed DGE, and among the 40 cases in which lymph node No. 3a was dissected, four cases (10%) developed DGE [89]. Of the five cases of DGE, three developed early postoperatively and had extended hospital stays; however, the subjective symptoms disappeared after discharge, and there was no recurrence of DGE. Two patients had persistent long-term DGE symptoms; of these, only one required additional surgical intervention because of long-term distress. An examination of the causes of DGE development revealed that gastric deformation was the primary cause. Considering these results, DGE appears to develop frequently after LR with limited lymph node dissection (approximately 10%), which is higher than that after LECS without lymph node dissection. However, gastric deformation, rather than denervation associated with lymph node dissection, appears to be the primary cause of DGE development.

**Figure 5:**
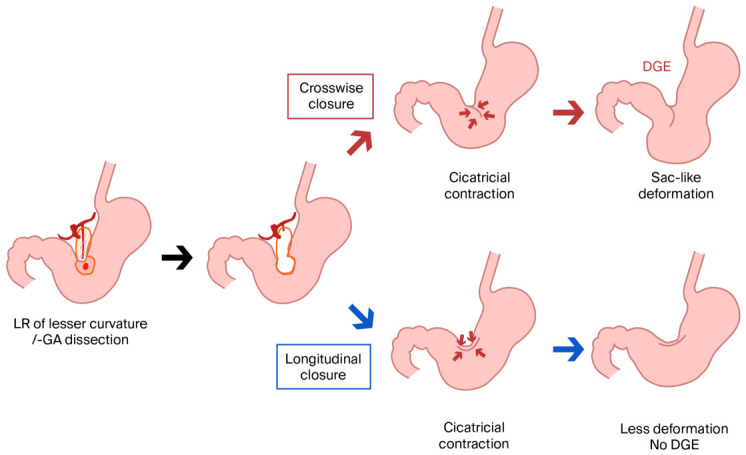
**Relationship between wound closure direction, gastric deformation, and the development of delayed gastric emptying: in cases of lesser curvature lesions.** Longitudinal closure results in less deformation and does not cause delayed gastric emptying (DGE). However, after cross-wise closure, cicatricial contraction can lead to sac-like deformation, potentially resulting in DGE. LR, local resection; l-GA, left gastric artery area.

**Figure 6:**
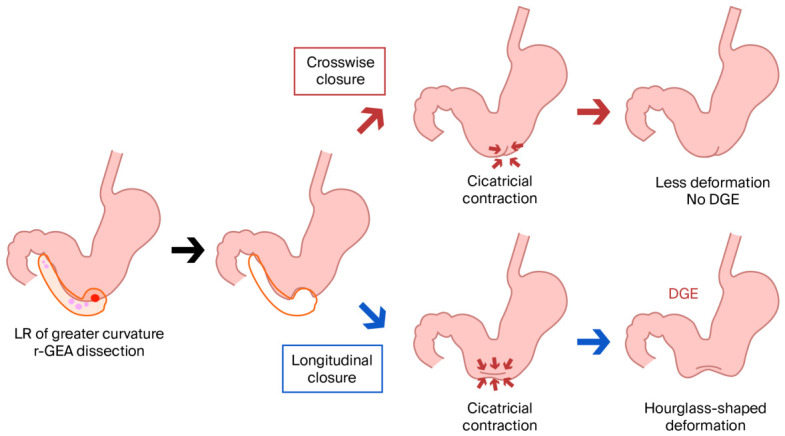
**Relationship between wound closure direction, gastric deformation, and the development of delayed gastric emptying: In cases of greater curvature lesions.** Crosswise closure results in less deformation and does not cause delayed gastric emptying (DGE). However, after longitudinal closure, cicatricial contraction can lead to hourglass-shaped deformations, potentially resulting in DGE. LR, local resection; r-GEA, right gastroepiploic artery area.

To prevent DGE after LR, sutures should be applied to minimize deformation [89]. To prevent sac-like and hourglass-shaped deformities, suture closure should be performed in the longitudinal or oblique direction after lesser curvature LR and suture closure should be in the short-axis direction after greater curvature LR (Figure 5 and Figure 6). Additionally, the addition of a pyloric finger boogie, similar to that used in PPG and segmental gastrectomy, may be an effective preventive measure against DGE.

## 7. Conclusions and Future Directions

Extensive gastrectomy leads to decreased food intake and weight loss and causes significant postgastrectomy complications. Since the lymph node metastasis rate of early gastric cancer is low, LR can be applied to reduce postgastrectomy syndrome without compromising survival prognosis in cases of solitary submucosal cancer less than 4 cm in size with no obvious lymph node enlargement on CT, and if metastasis is determined to be negative by SNB. However, because LR for early gastric cancer involves limited lymph node dissection, there is risk of developing DGE. To prevent DGE, adequate suture lines should be applied to minimize deformation.

Whether LR will become a standard treatment for early gastric cancer remains to be seen. Currently, it is difficult to apply this treatment approach without technology capable of performing a sentinel lymph node biopsy with high accuracy. A sentinel lymph node biopsy for gastric cancer is a complex procedure requiring a learning phase of 30 to 50 cases [13,14,46]. Submucosal cancers smaller than 4 cm that are not candidates for endoscopic submucosal dissection (ESD) are candidates for this procedure. However, they cannot be found without a medical checkup program that uses gastroscopy for cancer screening. Therefore, detection itself would be difficult in regions with a low incidence of gastric cancer, such as Europe and the United States. Most LR for early gastric cancer is currently only performed as an experimental treatment at a limited number of facilities in Japan and South Korea [46,47,48,49,50,51,52]. There are a few reports from Europe [90] or China [91]. Therefore, it is unclear if this method will spread beyond East Asia. Furthermore, there is currently no data on the cost-effectiveness of this treatment approach or on appropriate methods for residual stomach follow-up and on the diagnostic accuracy of identifying suitable cases. The proposed measures to prevent DGE in this article are currently only expert opinions, and their effectiveness requires further verification.

Nevertheless, LR for early gastric cancer is one of the few uncharted frontiers of surgical treatment. In the past, ESD for mucosal cancer was considered an unacceptable treatment due to its high level of difficulty, poor curative potential, and high incidence of metachronous multiple gastric cancers. However, advances in high-frequency electrosurgical devices, the development of specialized knife instruments, the verification of oncological safety through numerous prospective clinical trials, and the establishment of strategies for controlling metachronous multiple gastric cancers through Helicobacter pylori eradication therapy and re-ESD have made ESD a standard treatment [55,56,57,58,59]. Today, no one questions its legitimacy. For LR to become widespread, multicenter prospective trials will likely be essential [52]. Technological innovations such as novel tracers suitable for gastric cancer SNB [92,93,94], diagnosis of micrometastasis using fluorescent substances [95], and artificial intelligence applications in this field are expected to greatly reduce the current difficulty of gastric cancer SNB and LR. Within five to ten years, we believe LR will likely become part of the standard treatment for early gastric cancer in East Asia, and the guidelines will be rewritten.

## Figures and Tables

**Table 1:** Indications of local resection for gastric cancer.

Less than 4 cm in diameter. Having a clearly diagnosable border. Clinically diagnosed as submucosal cancer. No obvious nodal metastasis and distant metastasis in preoperative CT. Not in contact with the pylorus or cardia.

## Data Availability

This study contains no available external data.

## Abbreviations

The following abbreviations are used in this manuscript:

ESD	endoscopic submucosal dissection
LR	local resection
PPG	pylorus preserving gastrectomy
PG	proximal gastrectomy
LECS	laparoscopic endoscopic co-operative surgery
GIST	gastrointestinal stomal tumor
CT	computed tomography
MRI	Magnetic resonance imaging
SN	sentinel lymph node
SNB	sentinel node biopsy
SNFPG	sentinel node navigated function-preserving gastrectomy
SENORITA	SEntinel Node ORIented Tailored Approach
LSNNS	laparoscopic sentinel node navigation surgery
SNNS	sentinel node navigation surgery
ICG	indocyanine green
PGSAS	Postgastrectomy syndrome assessment scale
EORTC	European Organization for Research and Treatment of Cancer
DGE	delayed gastric emptying
TV	truncal vagotomy
SPV	selective proximal vagotomy
